# Identification of a Novel Hemizygous SQSTM1 Nonsense Mutation in Atypical Behavioral Variant Frontotemporal Dementia

**DOI:** 10.3389/fnagi.2018.00026

**Published:** 2018-02-06

**Authors:** Lin Sun, Zhouyi Rong, Wei Li, Honghua Zheng, Shifu Xiao, Xia Li

**Affiliations:** ^1^Shanghai Mental Health Center, Alzheimer's Disease and Related Disorders Center, Shanghai Jiao Tong University School of Medicine, Shanghai, China; ^2^Fujian Provincial Key Laboratory of Neurodegenerative Disease and Aging Research, Institute of Neuroscience, College of Medicine, Xiamen University, Xiamen, China; ^3^Department of Neuroscience, Shenzhen Research Institute of Xiamen University, Shenzhen, China

**Keywords:** Frontotemporal dementia, FTD, *SQSTM1*, p62, S224X, nonsense mutation

## Abstract

Frontotemporal dementia includes a large spectrum of neurodegenerative disorders. *SQSTM1*, coding for p62 protein, plays a vital role in the pathogenesis of FTD. Here, we report a case of a female patient with *SQSTM1* mutation S224X, who was 59 years old when she initially exhibited memory decline, mild personality changes, and subtle atrophy of frontal/temporal lobes in magnetic resonance imaging (MRI). Genetic testing revealed a nonsense mutation of the *SQSTM1* gene (S224X), resulting in premature termination of protein synthesis and a predicted truncated protein 217 amino acids shorter than the normal protein. Moreover, neither intact nor truncated SQSTM1 proteins was detectable in *SQSTM1* S224X mutant overexpressing HEK-293T cells. We assayed for *SQSTM1* cDNA in samples from the patient's peripheral leucocytes, and did not detect its mutation. The test of quantitative PCR showed significant decreased level of *SQSTM1* mRNA from peripheral leucocytes of the patient compared to five dementia controls. Our results identify a novel pathogenic *SQSTM1* S224X mutation in an atypical FTD patient accompanied with loss of SQSTM1/p62 protein expression probably due to *SQSTM1* gene haploinsufficiency.

## Introduction

Frontotemporal dementia is the second common form of neurodegenerative dementia in presenile population, characterized by atrophy of frontal and/or temporal lobes, and is frequently linked to genetic mutations (Miller et al., 2015; Luis et al., 2016). The three primary clinical FTD subtypes include behavioral variant FTD (bvFTD), nonfluent variant primary progressive aphasia (nfvPPA), and semantic variant primary progressive aphasia (svPPA) (Miller et al., 2015; O'Connor et al., 2016). As the most common presentation, bvFTD presents as a progressive change in personality with abnormalities in social-emotional behavior, and its diagnosis remains difficult, with patients being erroneously considered as having Alzheimer's disease or psychiatric disorders (Pottier et al., 2016; Tosun et al., 2016). The definite diagnosis is based on three major pathological subtypes characterized by the presence of 43 kDa TAR DNA-binding protein (TDP43), tau, or fused in sarcoma (FUS) positive neuronal inclusions (Boutoleau-Bretonniere et al., 2015). Approximately 30–50% of FTD patients have a positive family history, and 10% exhibit an autosomal dominant mode of inheritance. Mutations in the genes that encode microtubule associated protein tau (MAPT), progranulin and C9orf72 are the most common causes of FTD (Sun et al., 2017). Sequestosome 1 (SQSTM1), coding for p62 protein, is an adaptor protein that contains several protein-protein interaction motifs and serves as a signaling hub in a variety of key cellular processes including cell differentiation, transcriptional regulation, apoptosis, and oxidative stress response (Rea et al., 2014). SQSTM1, which has been identified in FTD in 2012 (Rubino et al., 2012), suggesting a role in the pathogenesis of neurodegenerative disease (Boutoleau-Bretonniere et al., 2015), is initially considered as a monogenic cause of Paget disease of bone (PDB) in 2002 (Laurin et al., 2002) and amyotrophic lateral sclerosis in 2011 (Fecto et al., 2011). Mutation in the *SQSTM1* gene is a rare cause of FTD and ALS (van der Zee et al., 2014). Rubino et al. only identified 3 missense mutations in the *SQSTM1* gene in 3 of 170 Italian patients with FTD, and 3 missense variants in 3 of 124 Italian patients with ALS (Rubino et al., 2012).

Here we report an atypical bvFTD patient with memory decline as an initial symptom and mild personality change emerging gradually, carried a novel pathogenic variant of the *SQSTM1* gene causing absent expression of SQSTM1/p62 protein.

## Materials and methods

### Genetic procedures

Total genomic DNA was prepared and amplified from peripheral blood according to standard procedures. The quality of DNA was assessed by Qubit 3.0 (Thermo Fisher, USA) and agarose gel electrophoresis. Then the sequencing library was prepared according to the SureSelectXT Target Enrichment System Manual (Agilent, USA), and whole exome sequencing was performed by HiSeq X Ten (Illumina, USA). After this next-generation sequencing and bio-information analysis of the sequencing data, especially AD and FTD related genes including *APP, PSEN1, PSEN2, MAPT, GRN, CHMP2B, C9ORF72, VCP, FUS, SQSTM1, TREM2, TYROBP* etc., we found that there was a nonsense mutation in *SQSTM*1 gene. The nonsense mutation of *SQSTM1* gene were analyzed by Sanger sequencing (forward primer: 5′-AGCGTCTGCCCAGACTACGA-3′ and reverse primer: 5′-CAGGCACTTAGGCACCTCAG-3′, the values of Tm were 63.4 and 61.0 respectively, and the length of amplified product is 547 bp). Moreover, we detected *APOE* genotyping at locus of rs429358 and rs7412. The software of Mutation Taster was applied to predict the pathogenicity of the detected mutations (http://www.mutationtaster.org/).

### Analysis of SQSTM1 mutant protein expression

Plasmid DNA for wild type *SQSTM1* was prepared by inserting coding sequence of the human *SQSTM1* gene (NM_003900) into the pcDNA3.1/Myc-His vector, and the *SQSTM1* S224X mutation was obtained by PCR-based site-directed mutagenesis with c.671C>A. All constructs were verified by sequencing (Minbo Biotech, Xiamen, China). Human embryonic kidney cells (HEK 293T) were grown to 80% confluence and transfected with vectors by Turbofect Transfection Reagent (Thermo Fisher, USA) according to the manufacturer's instructions. Media were then replaced with fresh DMEM containing 10% FBS. Cells were then collected 24 h later for western blotting. Equal amounts of total proteins (20 μg) were subjected to SDS-PAGE and transferred to PVDF membrane (Millipore, USA). Membranes were incubated with antibodies specific for SQSTM1 Gly162 (Cell Signaling Technology, 8025, USA), SQSTM1 Gly410 (Cell Signaling Technology, 5114, USA), Myc (Proteintech, 16282-1-AP, USA), or GAPDH (Abcam, ab181602, USA). Proteins were quantified using ImageJ Software.

### Characterization of SQSTM1 gene expression

The mutation S224X (c.671 C>A) of *SQSTM1* cDNA amplicons were obtained from the patient's mRNA sample by RT-PCR reaction. The cycling parameters of RT-PCR were 3 min at 95°C, followed by 30 s at 94°C, followed by 35 cycles of 60°C for 30 s, 72°C for 30 s, and a final extension of 72°C for 5 min. The pair of PCR primer sequence included the forward primer 5′-CTGTCTGAGGGCTCTCGC-3′ and reverse primer 5′-TCAACTTAATGCCCAGAGG-3′. The pyrosequencing and Sanger sequencing were performed following the manufacturer's protocols (Sangon, China). The analysis was used through PyroMark Software 1.0.11 software environment (Sangon, China).

Total cellular RNA was extracted from cell culture using TRIzol (Invitrogen, USA) according to manufacturer's procedures. Real-time PCR analysis was performed using 7900HT PCR instrument (ABI, USA). PCR conditions were at 95°C for 1 min, followed by 40 cycles at 95°C for 15 s, and 60°C for 30 s. For each biological replicate, three technical replicates were performed. The pair of PCR primer sequence included the forward primer 5′-TGGCGGAGCAGATG AGGAAG-3′ and reverse primer 5′-GGACTGGAGTTCACCTGTAGACG-3′.

### Statistical analysis

All data are presented as mean ± standard error of mean. The data were analyzed by one-way analysis of variance (ANOVA). Results were considered to be statistically different when *p* < 0.05.

### Ethics and patient consent

We received approval from the regional ethical standards committee on human experimentation for our experiments using human materials. We also received written informed consent for research from the participants and guardians.

## Results

### Case report

The patient underwent a clinical evaluation at our institution and was then enrolled in the Foundation of China Alzheimer's disease and related disorders study. Additional data from the proband and her relatives were collected and analyzed.

A 65-year-old, right-handed female with 15 years of school education was first seen in our geriatric psychiatry department in December 2016 for memory difficulties over 6 years combined with mild personality change over 1 year. She received surgical treatment for oophoroma in 2005 and drug treatment for hyperthyroidism in 2015, and gained full control of both diseases.

**Figure 1:**
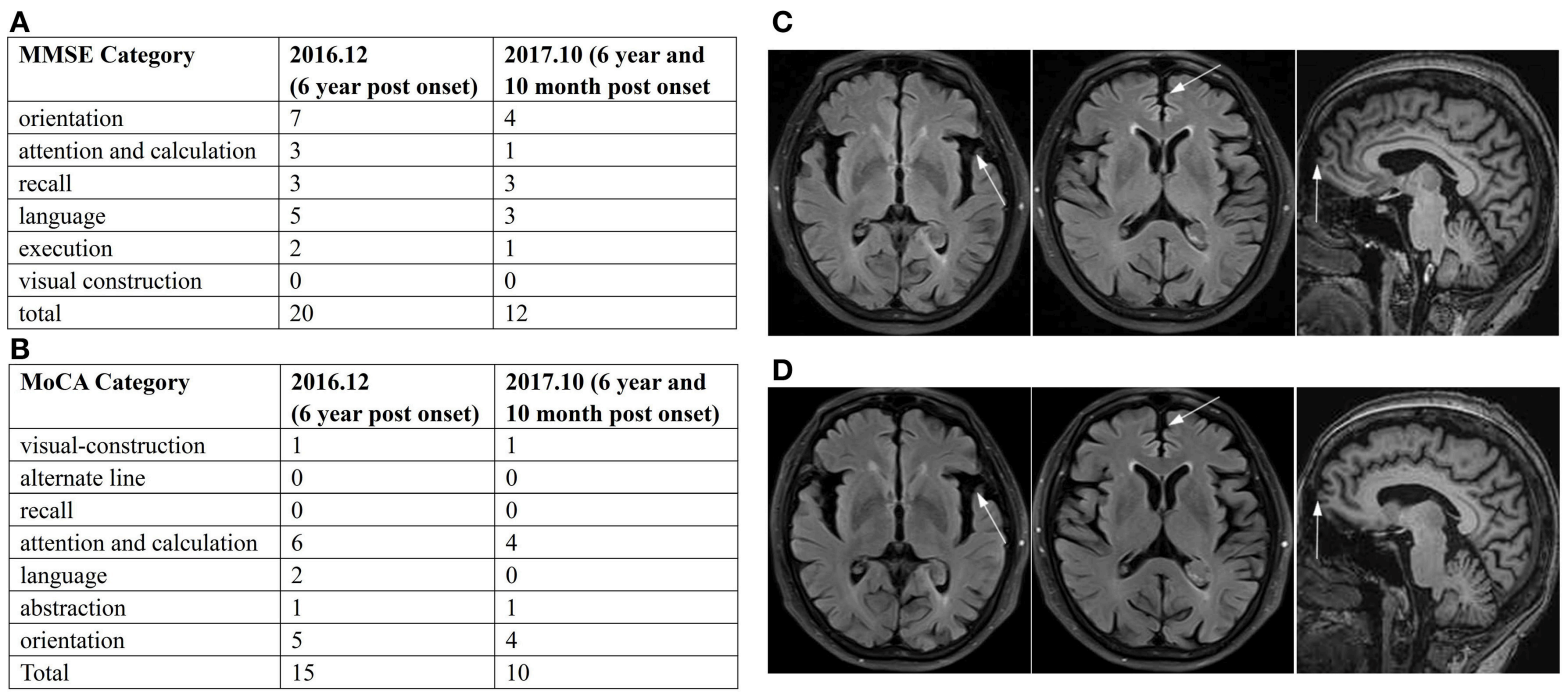
Summary of MMSE and MoCA, and imaging data. Summary of MMSE **(A)** and MoCA **(B)** scores in Dec 2016 and Oct 2017 displayed the significant damage in cognitive function and decline trend with time. The brain MRI in Dec 2016 **(C)** and Oct 2017 **(D)** showed subtle atrophy of frontal and temporal lobes on transverse FLAIR weighted and sagittal T1 weighted sequences.

Her caregiver described the patient's forgetfulness at age 59, which had developed insidiously. She was referred to hospital because of memory decline and depression, and was prescribed antidepressant drugs. The patient refused to take the medications, and went on to care for her sick mother. At 5 years post-onset of symptoms, she began to lose her way and fall occasionally. In June 2015, she fell, resulting in cracking her head and bleeding. Her family brought her to hospital for testing. The brain computed tomography (CT) revealed cerebral atrophy, and electromyogram/ nerve conduction velocity (EMG/NCV) showed no positive finding. Subsequently, therapy with huperzine A was initiated. However, the patient took the medication irregularly, and her condition gradually aggravated. In May 2016, her family noticed her daytime somnolence, sluggishness, and reticence. Magnetic resonance imaging (MRI) showed mild atrophy of the cerebral cortex. Subsequently, combination therapy with Exelon and Escitalopram was started. In December 2016, the patient was brought to our department, and standard blood tests were normal. The neurological evaluation showed slow gait, normal muscular tension, brisk tendon reflexes, positive sign of bilateral palm-chin reflex, and negative Babinski sign. Her Mini Mental State Examination (MMSE) score was 20/30 and Montreal Cognitive Assessment (MoCA) score 15/30 (Figures 1A,B). Brain MRI in Dec 2016 revealed subtle atrophy in frontal and temporal lobes (Figure 1C). Although these findings did not lead to a definitive diagnosis, Memantine, Exelon, and Sertraline were administered as therapy. After 10 months, she was referred to our department again, and complained more serious memory decline. Her MMSE score was 12/30, and MoCA 10/30 (Figures 1A,B). However, brain MRI in Oct 2017 revealed no significant difference when compared with the last MRI (Figure 1D).

**Figure 2:**
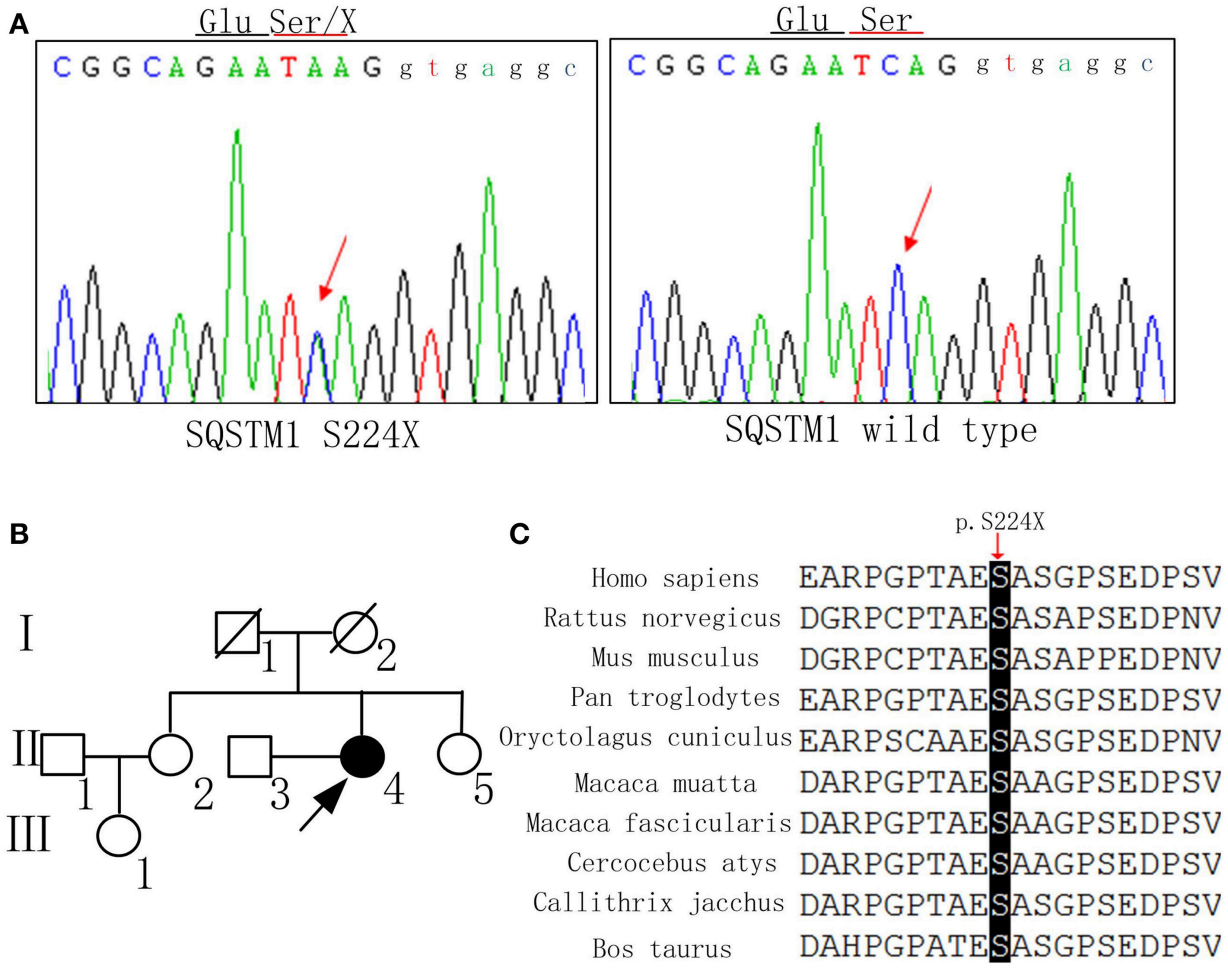
DNA sequence, pedigree of the family, and gene conservation. **(A)** DNA sequence at codon 224 of *SQSTM1* gene from the patient and a control. The arrow indicates a mutated hemizygous site and a normal homozygous site, respectively. **(B)** The proband is indicated by an arrow (II-4). Her younger sister (II-5) also received the gene test and did not carry the same mutation. **(C)** The p.S224X heterozygous nonsense mutation occurs at highly conserved position, as shown by a comparison of the corresponding sequences of 10 vertebrates.

The family history is summarized in Figure 2B. There are no similar manifestations in the relatives of the patient.

### Genetic analysis

The mutation c.671 C>A, p. S224X of *SQSTM1* was detected by whole exome sequencing, and validated in gDNA by Sanger Sequencing (Figure 2A). This mutation has not been reported as pathogenic elsewhere, and is predicted to lead to premature termination of protein synthesis. The pathogenicity prediction of the nonsense mutation by Mutation Taster software was disease causing with a probability equal to 1. The Combined Annotation Dependent Depletion (CADD) predicted that Raw score was 6.91 and PHRED was 33. The mutation was not found in the dbSNP, 1000G, HGMD, or ExAC database. At the same time, we didn't detect the same mutation in 200 normal Chinese individuals. The mutation site of the *SQSTM1* gene was also assessed by evaluating the patient's unaffected younger sister, who did not carry the same mutation. Because the patient's father and mother were deceased, we could not determine whether the mutation was hereditary. The p.S224X heterozygous nonsense mutation is located at highly conserved position, as shown by a comparison of the corresponding sequences of 10 vertebrates (Figure 2C). The *APOE* genotype of the patient was ε*3/*ε*4*.

### Analysis of SQSTM1 mutant protein expression

**Figure 3:**
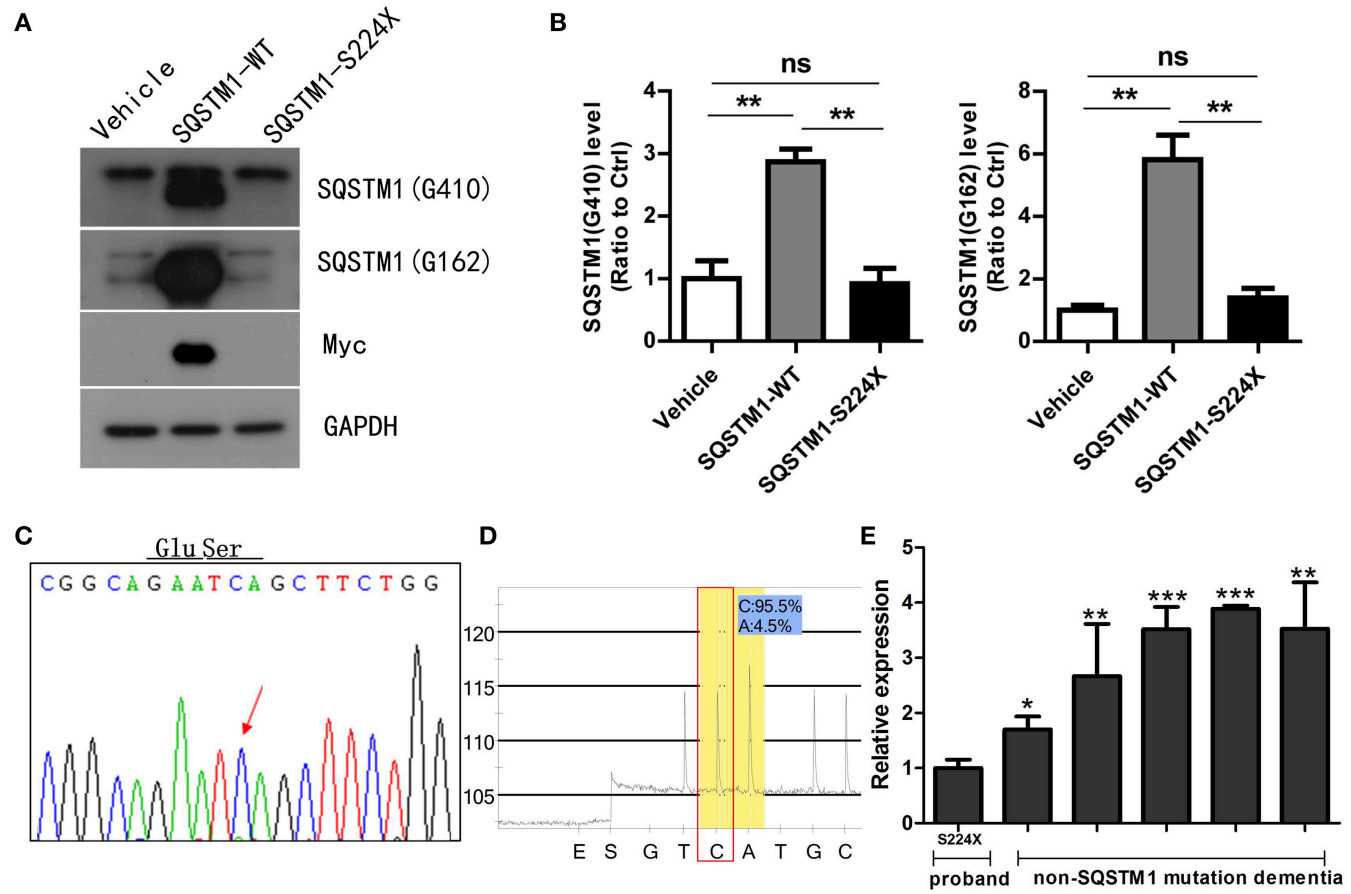
Mutant SQSTM1 protein and cDNA levels. **(A)** HEK 293T cells were transfected with an empty vector or plasmids encoding either wild type or mutant Myc-His-tagged *SQSTM1* (S224X) for 24 h. Intact and truncated SQSTM1 proteins were recognized by western blotting with SQSTM1 (G410) and SQSTM1 (G162) antibodies, respectively. **(B)** The protein levels of SQSTM1 were quantified by densitometry and expressed as ratios to GAPDH. There were significantly reduced levels of intact or truncated SQSTM1 proteins in the S224X mutation group compared to wild type, similar to the vehicle. There was no expression of Myc protein in the vehicle or S224X mutation group, only in wild type. Data were plotted as mean ± SEM (*n* = 3). ^**^p < 0.01, One-way ANOVA. **(C,D)** *SQSTM1* cDNA from peripheral blood leucocytes of the patient was analyzed by Sanger sequencing and pyrosequencing. The mutation was not detected in *SQSTM1* cDNA through Sanger sequencing, and only 4% of mutation was detected through pyrosequencing. The arrow indicates the mutant site. **(E)** *SQSTM1* gene expression, measured by quantitative real-time PCR, normalized to β-actin, in peripheral blood leucocytes from the patient and five dementia controls without *SQSTM1* mutation. The expression of *SQSTM1* mRNA from the patient was significantly reduced compared to controls. Data were plotted as mean ± SEM (*n* = 3). ^*^p < 0.05; ^**^p < 0.01; ^***^p < 0.001, one-way ANOVA.

HEK 293T cells were transfected with an empty-vector control or plasmids encoding either wild type or S224X mutant Myc-His tagged *SQSTM1* for 24 h. SQSTM1 was recognized in western blotting by different antibodies, which were SQSTM1 Gly162 and SQSTM1 Gly410 directed upstream and downstream, respectively, of the SQSTM1 mutation site (S224X). The SQSTM1 Gly162 antibody was used to detect truncated and intact SQSTM1 protein, and Gly410 to detect intact SQSTM1 protein (Figure 3A). Western blotting demonstrated that the levels of intact or truncated SQSTM1 protein in the S224X mutation group were all significantly reduced compared to wild type, similar to the vehicle (*p* < 0.01) (Figure 3B). Western blotting of Myc protein demonstrated no detectable Myc protein in the vehicle or S224X mutation group, only in the *SQSTM1* wild type group (Figure 3A).

### Analysis of SQSTM1 gene expression

*SQSTM1* cDNA was amplified by RT-PCR from mRNA extracted from peripheral blood leucocytes of the patient. We did not find the same mutation in *SQSTM1* cDNA (Figures 3C,D) through pyrosequencing or Sanger sequencing, which was inconsistent with DNA sequencing result. For analyzing the gene transcription differences between the *SQSTM1* S224X mutation and *SQSTM1* wild type, we included another five dementia controls (female: 3, male: 2, and age matched) without the *SQSTM1* mutation. The relative transcription of *SQSTM1* normalized to β-actin was analyzed using the concept of the threshold cycle (Ct) comparative method. Although the small sized of the samples adopted in the present study, it seemed that all dementia controls without *SQSTM*1 mutation presented an increased relative transcription level of *SQSTM1* mRNA (up to 3.06 ± 0.88-fold) with regards to the patient (*p* < 0.05) (Figure 3E).

## Discussion

The patient exhibited early-onset dementia combined with the initial symptom of memory decline, a gradual appearance of mild personality change, subtle atrophy of frontal/temporal lobes in MRI, negative appearance in EMG/NCV, and rapidly progressive course. The data of whole exome sequencing showed that AD related genes were all negative. These findings suggested an atypical bvFTD. The *SQSTM1* mutation (S224X) was found in the patient, which produced a stop codon and resulted in a predicted truncated protein 217 amino acids shorter than the normal SQSTM1 protein. *SQSTM1* has been reported to be associated with FTD-ALS type 3 (MIM: 616437). There isn't positive finding of EMG/NCV at present, but it still needs time to follow up the patient. The mutation is novel, not appearing in the dbSNP, 1000G, HGMD, ExAC database, and 200 normal Chinese individuals. The pathogenicity prediction by the Mutation-Taster application was disease causing, with a probability equal to 1. To determine the pathogenicity of this mutation, we overexpressed *SQSTM1* S224X mutant comparable to that of wild type *SQSTM1* in cell culture. Western blot of SQSTM1 recognized by Gly 410 and Gly 162 antibodies showed significant decrease of fully intact protein and truncated protein of SQSTM1. There was no expressional difference between mutant protein and vehicle. Western blotting of the Myc tag demonstrated that the S224X mutation led to loss of SQSTM1 protein expression after eliminating the effect of endogenous SQSTM1 protein from HEK 293T cells. Furthermore, in *SQSTM1* cDNA from peripheral blood leucocytes of the patient, we didn't detect the mutation (S224X) by Sanger sequencing and pyrosequencing, and found significantly decreased level of *SQSTM1* mRNA compared to five dementia controls without SQSTM1 mutation. The above results suggested absent expression of SQSTM1/p62 protein in the S224X mutant overexpressing HEK-293T cells and significant decrease of *SQSTM1* mRNA level in the patient, which was possibly caused by nonsense-mediated mRNA decay (NMD), an mRNA degradation pathway regulating gene expression and mRNA quantity (Lopez-Perrote et al., 2016).

The *SQSTM1* gene encodes SQSTM1/p62 protein, a scaffolding protein, which regulates a variety of biological processes, including nuclear factor kappa B (NF-κB) signaling, apoptosis, transcription regulation, and ubiquitin-mediated autophagy (Rea et al., 2014). Collet et al. demonstrated that PDB patients with *SQSTM1* mutation (P392L, A381V, A390X, and L413F) had an increased level of SQSTM1/p62, and overproduction of the protein probably was involved in the pathophysiology of PDB (Collet et al., 2007). In FTD patients, *SQSTM1* mutations (E396X and R212C) are reportedly associated with p62 and TDP43 inclusions in brain (Kovacs et al., 2016). However, in the present study, we demonstrated that a novel *SQSTM1* mutation (S224X) causing loss of SQSTM1/p62 protein expression, not protein overproduction. Haack et al. identified three different biallelic loss-of-function variants (c.2T>A, p.?; R96X; E104Vfs^*^48) in *SQSTM1* gene in nine patients with neurodegenerative disorder, and confirmed absence of SQSTM1/p62 protein in these patients (Haack et al., 2016). In mice, the knock out of *SQSTM1* led to obesity and impaired glucose tolerance (Rodriguez et al., 2006). Furthermore, the chronic absence of SQSTM1/p62 promotes neurodegeneration with neurofibrillary tangles in hippocampal and cortical neurons manifesting with depression and short-term memory decline (Haack et al., 2016), which is similar to the clinical presentations of the present patient. There are multiple variants of *SQSTM1* gene that cause diverse patterns of protein expression. Generally speaking, the imbalance of SQSTM1/p62 expression induced by *SQSTM1* mutations is probably involved in the pathological mechanisms of FTD.

SQSTM1/p62 plays a key role in a variety of vital cellular processes, but it is unexpected that its absence is compatible with survival above age of 40 years (Haack et al., 2016), and mice lacking SQSTM1/p62 were fertile and lived more than 1 year inspite of adult-onset obesity and diabetes (Komatsu et al., 2007). This phenomenon probably argues for a redundancy of involved pathways or effective compensatory mechanisms (Haack et al., 2016). The present patient likely reflected SQSTM1 gene haploinsufficiency due to a combination of protein instability and NMD. Meanwhile, the patient displayed the *APOE* ε*3/*ε*4* genotype, which confers an increased risk of developing Alzheimer's disease (AD). Whether APOE ε4 is a risk factor for FTD remains controversial. Ji et al. examined 432 patients with AD, 62 with FTD, and 381 controls. *APOE* ε*4* allele frequency was significantly increased in late-onset AD (24.86), early-onset AD (18.02), and FTD (16.13) patients compared with controls (7.34), which suggested that the ApoE ε4 genotype is a risk factor for AD and FTD (Ji et al., 2013). However, Gustafson et al. and Verpillat et al. reported no correlation between the ε4 allele and FTD, but a larger increase in the ε2 allele in FTD compared with controls (Gustafson et al., 1997; Verpillat et al., 2002). Whether the *APOE* ε*3/*ε*4* genotype in this case is promoting FTD requires further scrutiny.

In summary, we firstly identified a novel *SQSTM1* mutation (S224X) in an atypical bvFTD patient, and the mutation caused absence of SQSTM1/p62 protein, which was consistent with a reduced level of *SQSTM1* mRNA from peripheral leucocytes of the patient. The mechanisms underlying these observations are possibly associated with *SQSTM1* gene haploinsufficiency. Different variants of the *SQSTM1* gene result in diverse expression patterns, and imbalance of SQSTM1/p62 protein induces different pathogenic processes. In addition, there was a factor that limited the findings of the present study. Without a large family showing dementia and enough gene samples from family members, it is difficult to demonstrate that *SQSTM1* S224X was fully responsible for FTD. However, we have provided a clue for discussing the pathogenicity significance of *SQSTM1* S224X mutation in FTD, which should foster an understanding of the effect of *SQSTM1* mutation on FTD when the mutation can be verified in a large family showing dementia.

## Ethics statement

This study was carried out in accordance with the recommendations of “Shanghai Mental Health Center ethical standards committee on human experimentation” with written informed consent from all subjects. All subjects gave written informed consent in accordance with the Declaration of Helsinki. The protocol was approved by the “Shanghai Mental Health Center ethical standards committee”. All subjects also gave written informed consent for the publication of this case report.

## Author contributions

LS and ZR: performed the experiments; LS: wrote the paper; WL: collected the samples; XL, SX, and HZ: supervised the experiments.

### Conflict of interest statement

The authors declare that the research was conducted in the absence of any commercial or financial relationships that could be construed as a potential conflict of interest.
